# Asbestos Exposure and Severity of COVID-19

**DOI:** 10.3390/ijerph192316305

**Published:** 2022-12-06

**Authors:** Galo Granados, María Sáez-López, Cristina Aljama, Júlia Sampol, María-Jesús Cruz, Jaume Ferrer, Se-COVID-19 Team

**Affiliations:** 1 Department of Respiratory Medicine, Vall d’Hebron University Hospital, Passeig Vall d’Hebron, 119-129, 08035 Barcelona, Spain; 2 CIBER Enfermedades Respiratorias (CIBERES), Instituto de Salud Carlos III, 28029 Madrid, Spain; 3 Department of Respiratory Medicine, Universitat Autònoma de Barcelona (UAB), 08193 Barcelona, Spain

**Keywords:** COVID-19, asbestos exposure, occupational exposure, questionnaire (QEAS-7)

## Abstract

Background: The aim of this study was to analyse the relationship between occupational exposure to asbestos and the severity of SARS-CoV-2 infection. Methods: We evaluated patients who survived admission in our centre for COVID-19 pneumonia. Demographic, analytical, and clinical variables were collected during admission. After discharge, a previously validated occupational exposure to asbestos questionnaire was administered. Spirometry, CO diffusion test, the 6-min walk test, and high-resolution chest CT were performed. Patients who required respiratory support (oxygen, CPAP, or NIV) were considered severe. Results: In total, 293 patients (mean age 54 + 13 years) were included. Occupational exposure to asbestos was detected in 67 (24%). Patients with occupational exposure to asbestos had a higher frequency of COVID-19 pneumonia requiring respiratory support (*n* = 52, 77.6%) than their unexposed peers (*n* = 139, 61.5%) (*p* = 0.015). Asbestos exposure was associated with COVID-19 severity in the univariate but not in the multivariate analysis. No differences were found regarding follow-up variables including spirometry and the DLCO diffusion, the 6-min walk test, and CT alterations. Conclusions: In hospitalised patients with COVID-19 pneumonia, those with occupational exposure to asbestos more frequently needed respiratory support. However, an independent association between asbestos exposure and COVID-19 severity could not be confirmed.

## 1. Introduction

The COVID-19 pandemic has affected more than 480 million people all over the world, and more than six million have died. During the first wave, 15% of infected people required hospital admission and 4% died, mainly due to respiratory failure [1]. 

SARS-CoV-2 is an RNA virus that affects bronchial epithelial cells, type I and type II alveolar pneumocytes, and capillary endothelial cells by cleaving the receptor for angiotensin-converting enzyme 2 (ACE2) and by activating the S protein of the virus [2]. In severe cases, pneumonia occurs, and the worst prognosis is associated with the appearance of an inflammatory reaction in the form of respiratory distress [3]. The most severe patients also experience a prothrombotic state and interstitial fibrotic changes that may persist and cause a restrictive ventilatory disorder in survivors [4,5,6].

Advanced age and male sex are among the factors of severity of COVID-19, and the main comorbidities are obesity, diabetes, and high blood pressure [7,8,9]. In addition, pulmonary inflammation in severe cases correlates with alterations in analytical parameters such as d-dimer, neutrophils, C-reactive protein (CRP), and ferritin [10].

The influence of exposure to inhaled agents on the severity of COVID-19 has received less attention. More severe COVID-19 infection has been described in areas with high environmental contamination [11,12] and a recent epidemiological study in England demonstrated an association between customer-facing and healthcare jobs with mortality due to COVID-19 [13].

Inhalation of asbestos fibres is a known cause of neoplastic and fibrogenic lung injury and pleural diseases such as asbestosis, a form of pulmonary interstitial pneumonia [14,15]. Asbestos increases oxidative stress and activates a persistent low-grade inflammatory response that favours the development of diseases after a latency period of more than 15 years [16,17,18,19]. This sustained inflammation has also been observed in people exposed to asbestos who are not yet ill [16]. In exposed subjects, activation of the inflammasome has been described with increases in various serum markers such as interleukin 1B, IL-18, TNF alpha, and RANTES [20], as well as a dysregulation of the immune system that predisposes one to chronic inflammation and carcinogenesis [21].

This study explores the hypothesis that asbestos exposure may be related to severe forms of COVID-19. The aim is to analyse occupational exposure to asbestos in patients who have survived admission for COVID-19 in order to establish whether they have a more severe form of the disease, defined as the need for respiratory support.

## 2. Materials and Methods

### 2.1. Study Population

**Figure 1:**
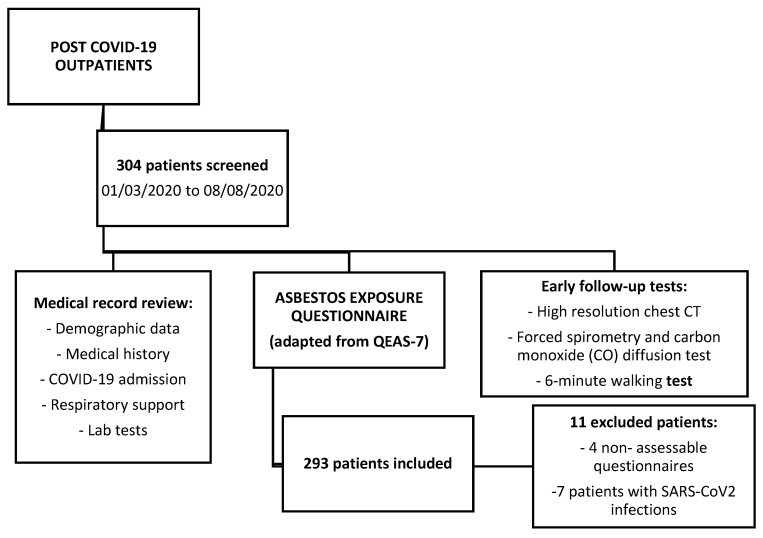
Flow chart of the patients recruited for the study.

Prospective observational study. A total of 304 patients were consecutively evaluated at the post-COVID-19 pulmonology outpatient offices of a university hospital in Barcelona between June and October 2020. All patients had been admitted to the centre due to COVID-19 pneumonia between 1 March and 8 August 2020. Figure 1 shows a flow chart tracing the inclusion process. The study was approved by the Ethics Committee of our centre (PR identification code (AG 387/220) and all participants gave written consent prior to recruitment.

### 2.2. Assessment of Asbestos Exposure 

During the first follow-up visit, all patients completed an asbestos exposure questionnaire, adapted from a previously validated checklist focusing on occupational exposure (QEAS-7) [22]. The questionnaire records respondents’ employment histories and includes a question about the use of asbestos in the workplace. In addition to this question, each participant had to review a list of 48 occupations or activities and another list of 70 materials that present a risk of exposure to asbestos, coded as red (high risk) or black (moderate risk), (see Supplementary Materials), and record any possible activity or contact with risk materials during their working life.

The responses to the questionnaires were assessed by two of the authors to establish the probability of occupational exposure to asbestos, which were classified into two categories: certain/probable or non-existent, according to the following criteria [22]:

- Occupational exposure was considered certain when a respondent gave an affirmative answer to question 1 regarding the use of asbestos in general, and when he or she ticked at least one activity or material from the lists. Exposure was also considered certain when a respondent gave a negative answer to question 1 but ticked a material or activity from the lists with a high risk of exposure.
- Occupational exposure was regarded as probable when a respondent answered question 1 in the affirmative but did not acknowledge any of the activities or materials listed. It was also considered probable in the case of an affirmative answer to a material or activity with a moderate risk of exposure.
- Occupational exposure was considered non-existent when a negative answer to question 1 was recorded along with negative answers to the items in the lists of materials and activities. It was considered unknown when the patient was unable to answer either question 1 or the lists of activities and materials.

### 2.3. Demographic and Clinical Data

The following demographic variables were recorded: sex, age, body mass index, and tobacco use with cumulative exposure in packs/year. Comorbidities were also recorded, grouped into cardiological, neurological, and psychiatric, as well as pulmonary history and diagnosis of diabetes mellitus.

The following data were recorded on admission: serum leukocytes, platelets, d-dimer, lactate dehydrogenase (LDH), C-reactive protein (CRP), and interleukin-6 (IL-6).

Patients were classified according to the severity of their COVID-19 pneumonia. Patients who did not need respiratory support were classified as mild-moderate, whereas those requiring respiratory support of any type, including conventional or high-flow oxygen therapy, CPAP, or non-invasive mechanical ventilation, were considered severe.

The variables obtained in the follow-up visit were the presence of dyspnea classified by the mMRC scale, spirometry including forced vital capacity (FVC), forced expiratory volume in the first second (FEV_1_) and FEV_1_/FVC ratio, carbon monoxide (CO) diffusion test, and the total distance walked in the 6-min walking test.

A high-resolution computed tomography (CT) chest scan was performed, and the pulmonary findings were classified into two grades according to the degree of involvement: pathological, which included the existence of alveolar or interstitial alterations or bronchiectasis, and non-pathological.

### 2.4. Statistical Analysis

Descriptive data are presented as n (percentage) or mean (SD). Comparisons according to the need for respiratory support were made using the Student’s *t*-test for quantitative variables and the Pearson chi-square test for categorical variables.

Logistic regression models were used to assess the characteristics associated with the development of severe COVID-19. Asbestos exposure was included in all models and covariates were selected prior to analysis. A first logistic regression model shows the effect of asbestos exposure without adjusting for any covariates. In a second model, we used a multivariate logistic regression including, apart from asbestos exposure, age and sex, which are factors that are associated with both severe COVID-19 (7) and asbestos exposure. We also used a multivariate logistic regression in a third model to calculate the Odds ratio associated to asbestos exposure adjusted by age and sex and the number of comorbidities that have been previously reported as related to severe COVID-19 disease (hypertension, diabetes, obesity, chronic respiratory disease) (7). A level of significance of 0.05 was used. 

The analysis was carried out using the statistical package Stata IC 14 (StataCorp. 2015. Stata Statistical Software: Release 14. StataCorp LP, College Station, TX, USA). 

## 3. Results

A total of 304 patients were evaluated, and 293 were finally included in the study (Figure 1). There was a slight predominance of men (53.9%), with a mean age of 58.4 ± 12.8 years. The mean time between hospital discharge and the first outpatient visit was 111 ± 43 days. Sixty-seven patients (23.9%) were considered to have been exposed to asbestos, with a mean duration time of 17.5 years (SD: 11.3). The occupations of all the patients and of those exposed to asbestos are shown in Table 1. In the overall set of patients, the most common occupations were commerce and the food industry, housework, cleaning, and construction. Construction workers, artisans, and workers in the rubber and plastic industry predominated in the group of patients exposed to asbestos.

The relationships between occupational exposure to asbestos and the main variables studied during and after hospital admission are shown in Table 2 and Table 3, respectively. The most notable result was the significantly higher percentage of patients requiring respiratory support among those exposed to asbestos. Other more frequent variables in the exposed patients were older age, predominance of males, smoking, and a history of diabetes and cardiological and respiratory pathologies. Asbestos exposure was associated with severe COVID-19 in the univariate analysis, but this hypothesis could not be confirmed in the logistic multivariate regression analysis (Table 4). 

Regarding the variables related to disease progression, exposed patients presented a more intense degree of dyspnea, with the difference almost reaching statistical significance. No differences were detected in spirometry parameters except for a lower FEV_1_/FVC in the asbestos exposure group. There were no differences in the rest of the variables relating to the respiratory function or in the percentage of patients with lung lesions on chest CT. 

## 4. Conclusions

This study shows that in hospitalised patients who have survived COVID-19 pneumonia, the proportion of severe cases requiring respiratory support is higher in those with occupational exposure to asbestos than in their unexposed peers. However, asbestos exposure was not independently associated with COVID-19 severity in the multivariate analysis. Similarly, in a previously reported series of patients with severe COVID-19, exposed patients were older, more frequently male, smokers, and had more comorbidities. Thus, confounding by these covariables may have precluded the detection of an independent association in this relatively small sample of patients. 

To date, the study of the relationship between exposure to exogenous agents by inhalation and the severity of COVID-19 has focused on environmental contamination [12]. It has been shown that there is a direct relationship between geographical contamination before the pandemic and the incidence of COVID-19, hospital admission, and mortality caused by the infection. These results have been obtained in studies of different populations in China, America, and Europe, including our own region in Spain [23,24,25,26,27,28]. Regarding the possible association between occupation and COVID-19, several studies have shown a higher risk of infection and mortality due to COVID-19 in essential workers such as health care staff and teachers and have highlighted the risk of disease transmission in sectors such as health services [29]. In the most recent study, carried out in England with an epidemiological design, customer-facing workers and health professionals were associated with the highest risk of death from COVID-19, although the authors admitted some limitations in their study such as the use of a previous occupational history and the existence of confounding factors such as geographical location, socioeconomic level, or lifestyle [13]. To the best of our knowledge, the present study is the first to investigate a relationship between the severity of COVID-19 and occupational exposure, specifically to asbestos, determined prospectively using a specific validated questionnaire.

The hypothetical contribution of asbestos exposure to the severity of COVID-19 might occur in different ways. Inhaled fibres are partially eliminated by the mucociliary barrier and partially phagocytosed by alveolar macrophages and translocated to lymph nodes. Despite the action of these clearance mechanisms, pulmonary deposition is frequent. The fibres retained in the lung can activate a low-grade inflammation that likely promotes the fibrogenic and carcinogenic changes typical of the asbestos-related diseases [30]. In exposed subjects, the activation of intracellular signalling pathways such as tyrosine kinase, which stimulates cell proliferation, carcinogenesis, and pulmonary fibrotic changes, has been described [31]. In an experimental murine model, inhalation of asbestos fibres induced the activation of the transcription factor NF-kAPPA-b in epithelial cells [32], a factor that also plays a central role in the activation of the inflammatory cascade after infection by SARS-CoV-2 [33]. After inhalation of asbestos, the NLRP3 inflammasome is activated in macrophages, monocytes, and epithelial cells with increases in IL-1B and IL-18. In workers exposed to asbestos, increases in serum levels of IL-1β, IL-6, IL-8, and TNF-α have been reported [20]. In addition, exposure to asbestos has been found to decrease immunity in those affected [34]. T cells from patients with mesothelioma stimulated by CD4 + lymphocytes in vitro showed a lower expression of interferon [35]. 

This previous evidence suggests that people exposed to asbestos might have an increased COVID-19 severity due to their immune alteration and an underlying inflammatory state. However, our results do not support this hypothesis. Moreover, we did not find a relationship between exposure to asbestos and a higher number of respiratory sequelae in the form of dyspnea or changes in spirometry or chest CT. This result argues against the possibility of a synergistic relationship between asbestos exposure and SARS-CoV-2 infection in the evolution towards pulmonary fibrogenesis.

A quarter of the patients admitted for COVID-19 had worked in occupations with a risk of exposure to asbestos. This percentage is lower than that of a previous study in Spain, in which 42% of patients presented exposure [36]. This difference may be due to the time elapsed between the two studies (22 years). The ban on the use of asbestos in Spain was introduced in 2001 and the average age of our unexposed subjects was 54 years, and so most of them had carried out their working lives after the ban. In addition, the percentage of patients who had worked in industrial or construction activities was only 16%, a circumstance that may explain the low detection of asbestos exposure despite the use of a questionnaire as sensitive as the QEAS-7.

In the present study, the need for some type of respiratory support during hospital admission was considered as an indicator of severity. The criterion used at our centre to decide hospital admission for most patients is the existence of pneumonia, as in most other hospitals in Spain. Pulmonary involvement can cause respiratory failure which is the reason for the indication of respiratory support with oxygen, through the use of various devices such as conventional masks, high-flow systems, or coupled to CPAP or non-invasive mechanical ventilation [37]. Accordingly, the definition used in this study to classify the most severe patients was the need for any type of respiratory support.

This study has several limitations. First, the study cohort included only patients who survived COVID-19 after hospital admission. Information on patients’ employment does not appear regularly in the medical records, and for this reason, it had to be obtained through the QEAS -7 questionnaire administered after recovery; as a result, we were unable to assess the effect of asbestos exposure on mortality. Second, another limitation of the study is its observational and single-centre design, which means that our results cannot be generalised to other settings. Furthermore, the presence of interacting variables may explain the lack of association found between asbestos exposure and severity of COVID-19. However, our cohort’s demographic and clinical characteristics and the set of variables related to the severity of COVID-19 are consistent with previous reports [3,38] underlying the interest of the findings.

Our study also has strengths. The prospective design made it possible to evaluate exposure to asbestos through a specific, previously validated structured questionnaire for its detection. In addition, the systematic evaluation of patients after hospital discharge also allowed for an assessment of the relationship between asbestos exposure and the respiratory sequelae of COVID-19. 

In conclusion, our study shows that the severe forms of COVID-19 are more frequent in patients with occupational exposure to asbestos than in their unexposed peers. However, our data could not establish the asbestos exposure as an independent risk factor for COVID-19 severity. In countries like ours, workers exposed to asbestos undergo regular check-ups at specialised centres. After this first study, further investigation could be useful to determine the exact nature of the relationship between asbestos exposure and severity of COVID-19. In view of our results, it would seem that patients exposed to asbestos without asbestos-derived diseases are not at a higher risk for severe COVID-19 than the general population.

## Figures and Tables

**Table 1:** Patients’ occupations.

**1a. Patients’ Occupations**	***n***	%
Food industry	56	19.11
Household and cleaning	55	18.77
Construction industry	39	13.31
Office work	27	9.22
Social sciences and law	18	6.14
Drivers	16	5.46
Health staff	16	5.46
Arts and crafts	14	4.78
Security and civil protection	14	4.78
Mechanics and painters	10	3.41
Textile industry	9	3.07
Education	7	2.39
Chemicals industry	6	2.05
Other	6	2.05
TOTAL	293	100.00
**1b. Occupations of patients exposed to asbestos ^a^**	***n***	%
Construction industry	33	49.3
Arts and crafts	9	13.45
Chemicals industry	4	5.97
Textile industry	6	8.95
Mechanics and painters	7	10.45
Drivers and vehicle repair	2	2.98
Other	6	8.9
TOTAL	67	100.00

^a^ Patients with more than one type of occupation are classified under the one they have carried out for the longest time period.

**Table 2:** Hospital admission data of patients according to occupational exposure to asbestos ^a^.

	Not Exposed *n* = 226	Exposed *n* = 67	*p*
Age	57 (12.9)	63.1 (11.4)	<0.001
Sex (male)	100 (44.2%)	58 (86.6%)	<0.001
BMI (kg/m^2^)	28.4 (5.1)	28.9 (4.4)	0.5463
Smoker	53 (23.4%)	30 (44.8%)	0.002
**Respiratory support**			
O_2_, MV, CPAP	139 (61.5%)	52 (77.6%)	0.015
**Prior medical conditions**			
Diabetes mellitus	28 (12.4%)	15 (22.4%)	0.042
Dyslipidemia	58 (25.7%)	25 (37.3%)	0.063
Cardiological	97 (42.9%)	38 (56.7%)	0.047
Neurological	17 (7.5%)	3 (4.4%)	0.385
Psychiatric	28 (12.4%)	3 (4.4%)	0.072
Respiratory	35 (15.5%)	19 (28.4%)	0.017
**Lab tests on admission**			
Leukocytes (x10E9/L)	7662 (5141)	8300 (4993)	0.3781
Platelets (x10E9/L)	300,888 (145,052)	276,323 (133,008)	0.2237
D-dimer (ng/mL)	1760 (4187)	2241 (5894)	0.4739
LDH (UI/L)	389 (173)	387 (148)	0.9252
PCR (mg/dL)	12.4 (14.2)	11.9 (9.4)	0.7782
IL6 (pg/mL)	470 (1570)	459 (1483)	0.9607
**Characteristics of admission**			
Complications	69 (30.5%)	28 (41.8%)	0.085
Length of stay (days)	14.5 (15.9)	15.3 (16)	0.7286

^a^ Data are presented as mean (SD) in quantitative variables or *n* (%) in categorical variables.

**Table 3:** mMRC dyspnea scale, lung function, and chest CT after hospitalization ^a^.

	NOT EXPOSED (*n* = 226)	EXPOSED (*n* = 67)	*p*
**mMRC dyspnea scale**			
0	130 (57.5)	40 (60.6)	0.050
1	67 (29.7)	13 (19.7)	
2	22 (9.7)	13 (19.7)	
3	7 (3.1)	0	
**Lung function tests**			
FVC L	3.5 (1)	3.7 (1)	0.1455
FVC %	97 (18.9)	94.9 (20.3)	0.4391
FEV1 L	2.9 (0.9)	2.9 (0.8)	0.6722
FEV1 %	99.1 (20.1)	96 (21.5)	0.2694
FEV1/FVC %	80.9 (7.3)	78.3 (8.1)	0.0133
DLCO %	76.9 (20.1)	75.4 (18.2)	0.5850
KCO %	83.7 (15.2)	84.6 (16)	0.6810
WT6 (m)	412 (95)	428 (84)	0.2511
**Chest CT**			
Lung sequelae	120 (53.3)	41 (61.2)	0.256

^a^ Data are presented as mean (SD) in quantitative variables or *n* (%) in categorical variables.

**Table 4:** Logistic regression analysis.

	Outcome: Severe COVID-19	
	OR	*p*
Asbestos exposure *	3.467 (1.952–6.157)	<0.001
Asbestos exposure †	1.288 (0.645–2.57)	0.473
Age †	1.018 (1.011–1.026)	<0.001
Sex †	0.416 (0.251–0.690)	0.001
Asbestos exposure ‡	1.159 (0.573–2.348)	0.681
Age ‡	1.012 (1.004–1.020)	0.005
Sex ‡	0.400 (0.238–0.671)	0.001
Comorbidity ‡	1.653 (1.190–2.296)	0.003

* Model 1: without adjustment. † Model 2: adjusted with age and sex. ‡ Model 3: adjusted with age, sex and number of comorbidities (hypertension, diabetes, obesity, chronic respiratory disease).

## Data Availability

Not applicable.

## Supplementary Materials

The following supporting information can be downloaded at: https://www.mdpi.com/article/10.3390/ijerph192316305/s1. Adaptation of Asbestos Exposure Questionnaire (QEAS-7) for Clinical Practice.
