# Supplementary material for: Asbestos Exposure and Severity of COVID-19
Source: Int J Environ Res Public Health. 2022 Dec 6;19(23):16305. doi: 10.3390/ijerph192316305 (PMC9739528; doi:10.3390/ijerph192316305)
Supplement: Supplementary file 1 [file ijerph-19-16305-s001.zip › ijerph-2010212-supplementary.pdf]

SUPPLEMENTAL MATERIAL

Abbreviated asbestos questionnaire
Adapted from a previously validated checklist focusing on occupational exposure (QEAS-7)

Subject's nº: .... Interviewer:...................................

Date of interview:........... /.......... / ....

1.Write the work activities you have performed, indicating the year of the start and end date.

WORK ACTIVITIES	START	END
1,		
2.		
3,		
4.		
5.		
6.		
7.		

2. In any of the jobs you have had, have you ever performed any activities that involved asbestos?

1) Yes (please specify) ……………………………………………………………
2) No/Don't know ………………………………………………………………

(If the answer is affirmative for more than one job, check that they are listed in the brief employment history and indicate the reference of the job(s) given an affirmative answer).

3. In any of the jobs you have had, have you ever carried out any of the activities or operations in List A? (make an X if the answer is "YES" and in the next column indicate the approximate time in years).

**List A: Activities or lobs of risk**	**YES**	**TIME (years)**
A.1. Shipyard workers (construction, repair or scrapping of boats)		
A.2. Workers in warehouses storing construction materials		
A.3. Sales representatives working with materials containing asbestos (brakes, clutches,		
thread, rope, cord, fabric, gaskets, cardboard, cardboard gaskets,…)		
A.4. Manufacturers of friction material (brakes, clutches)		
A.5. Manufacturers of fiber cement materials (uralite)		
A.6. Manufacturers of textiles containing asbestos (thread, rope, cord, fabric, gaskets,		
cardboard, asbestos fabric)		
A.7. Repairers of friction material (brakes, clutches)		
A.8. Farming (repair or maintenance of vehicles or machines)		
A.9. Workers in insulation or insulating coatings		
A.10. Masons (installation of fiber cement roofs or covers (plates or tiles)		
A.11. Graphic artists		
A.12. Craftsmen and manual and technical activities		
A.13. Firefighters		
A.14. Carpenters, formworkers, assemblers or cabinetmakers		
A.15. Drivers who carry out vehicle maintenance		
A.16. Electricians		
A.17. Workers in production or maintenance of train or subway cars		
A.18. Workers in production or repair of stoves		
A.19. Workers in production of paint		
A.20. Plumbers or pipe inspectors		
A.21. Smelters		
A.22. Workers in the food and beverage industry (repair or maintenance)		
A.23. Workers in ceramics (repair or maintenance)		
A.24. Workers in the plastic or rubber industry (repair or maintenance)		
A.25. Workers in the glass industry		
A.26. Kitchen installers		
A.27. Jewelers		
A.28. Chimney sweeps		
A.29. Workers in rehabilitation, repair or maintenance of industrial buildings		
A.30. Workers in rehabilitation, repair or maintenance of fiber cement materials (uralite)		
A.31. Car mechanics		
A.32. Elevator mechanics		

List A: Activities or lobs of risk	YES	TIME (years)
A.33. Mechanics in industry		
A.34. Workers in asbestos mines		
A.35. Dock workers		
A.36. Painters of buildings		
A.37. Workers repairing industrial machines		
A.38. Workers in the repair or maintenance of municipal water distribution networks		
A.39. Workers repairing electric motors		
A.40. Workers repairing and painting of car bodies		
A.41. Boiler room workers		
A.42. Workers in gas, nuclear and power plant services		
A.43. Military service and armed forces (repair or maintenance)		
A.44. Welders (also brass and tin makers)		
A.45. Ragpickers		
A.46. Roofers. Installation or repair of roofs or facades		
A.47. Ventilation technicians and air conditioning installers		
A.48. Textile workers (heat-resistant clothing)		

4. In any of the jobs you have had, have you ever carried out any activities or operations in List B? (make an X if the answer is "YES" and in the next column indicate the approximate time in years)

List B: Materials that contain asbestos (MCA)	YES	TIME (years)
(This list is not exhaustive; other products and materials may be added)		
MCA in construction materials		
The most widely used asbestos material is fiber cement or asbestos-cement, which has been applied		
in the construction industry in the form of:		
B.1. Corrugated plates on roofs and rain walls		
B.2. Flat plates on facades (rain walls) and balconies		
B.3. High pressure water pipes		
B.4. Drainpipes		
B.5. Water tanks		
B.6. Plant pots		
B.7. Exhaust ducts, chimneys and shunts		
B.8. Air conditioning ducts		
B.9. Fences for gardens and livestock		
B.10. Benches and outdoor tables		
Other forms of presentation of asbestos in construction are:		
B.11. Coating for the protection of metallic structures and as thermo-acoustic coating		
B.12. Asbestos fibers, used as interior insulation of fire doors in air chambers in walls and ceilings		

**List B: Materials that contain asbestos (MCA)**	**YES**	**TIME (years)**
B.13. Acoustic and thermal insulation		
B.14. Mortars for the protection of metallic structures		
B.15. Plates of varying densities for soundproofing		
B.16. Ceiling plates		
B.17. Mixed with paint and putty, it was used as a thixotropant; it also provided flame-retardant characteristics.		
B.18. Mixed with plastic material for vinyl flooring		
B.19. Mixed with other materials imitating wood, in interior decoration		
B.20. Adhesives and glues		
MCA in machines and installations		
As a textile material, asbestos may be found in:		
B.21. Blankets and felts for lining pipes		
B.22. Braids used to wrap pipes in order to isolate them		
B.23. Flame retardant fabrics for fire protection. Blankets, theater curtains, clothes		
B.24. Asbestos thread and cord		
B.25. Industrial and commercial felts		
B.26. Electrical insulation material		
B.27. Thermal protective clothing: gloves, clothing, aprons, etc.		
B.28. Gaskets		
As cardboard, asbestos can be found in:		
B.29. Cardboard or low-density plates to protect metallic structures or sources of heat		
(kitchens, fireplaces, etc.)		
B.30. Air conditioning ducts		
B.31. Flame-retardant absorbent paper		
B.32. Catalytic filters and insulation devices in heating appliances that use liquefied gas		
B.33. Protection for handling molten glass		
B.34. Corrosive gas and vapor lines		
B.35. Anti-heat and flame retardant components		
B.36. Insulating products		
B.37. Coating of electric motors to protect them from sources of heat		
B.38. Sealing gaskets		
As friction material		
B.39. Brake pads		
B.40. Drum brakes		
B.41. Clutches/transmission components		
B.42. Industrial friction material		
Other applications:		
B.43. Wire drawing		
B.44. Coating of glassware clamps		
B.45. Filters for beverages (beer and wine) and oils		
B.46. Filters for the transportation, distribution and use of gas		
B.47. Coating of clamps for crucibles		

**List B: Materials that contain asbestos (MCA)**	**YES**	**TIME (years)**
B.48. Thermal insulation in distillation columns		
B.49. Road surfacing material		
B.50. Fixing and joints for tiles		
B.51. Plastics and rubbers reinforced with asbestos		
B.52. Plastic components for electric motors		
B.53. Plastic components for molded products		
B.54. Sealing gaskets		
B.55. Electrical insulation parts with resins		
B.56. Filling of acetylene bottles		
MCA used in the household		
B.57. Thermal plate stoves		
B.58. Electric resistance stoves		
B.59. Kitchens with electric heating elements		
B.60. Electric heaters		
B.61. Butane gas heaters		
B.62. Ironing board covers		
B.63. Hair, feet and hand dryers		
B.64. Toasters		
B.65. Heat-resistant oven gloves		
B.66. Heat protection for formica furniture below ovens		
MCA in other applications or uses in laboratories (companies, institutes, technical schools,		
universities)		
B.67. Grids for heating glass appliances		
B.68. Internal insulation linings of muffle stoves and furnaces		
B.69. Pincers to hold crucibles		
